# Diagnosis under Artificial Light. Its Difficulties and a Solution of the Problem

**Published:** 1908-10

**Authors:** A. Cressy Morrison

**Affiliations:** Chicago; Member Illuminating Engineering Society


### Diagnosis Under Artificial Light. Its Difficulties and a Solution of the Problem.

By A. CRESSY MORRISON, Chicago.
Member Illuminating Engineering Society.

MOST physicians recognise the difficulty of accurately judging the condition of tissues by means of artificial illuminants. The difficulty, has however, usually been accepted as inevitable. Until recently, no artificial illuminant has been known which has the balanced spectrum of sunlight, and so where dignosis has been necessary by means of artificial illuminants, the physician has done the best he could, occasionally correcting his opinion after a verification of the examination by daylight.

The eye, through all ages, has adapted itself to see by daylight. Its evolution has been wrought in harmony with the solar spectrum. The seven colors of the solar spectrum have a given intensity; combined, they make white light. All artificial illuminants, save acetylene alone, have an excess of one color or another. City gas light has an excess of red, kerosene an excess of yellow and red, the Welsbach burner an excess of green, the arc light an excess of violet, the incandescent an excess of orange and red. Acetylene, on the other hand, has the seven colors in such intensity that, compared with sunlight, the difference is negligible. The excess of one color or another in artificial illuminants upsets the judgment of the physician, and is bound to have an effect upon the validity of a diagnosis. If the eye has adjusted itself to see best by daylight, then, under daylight, colors have their normal values, and the mind, trained and adjusted to base its conclusions upon what the eye reports, can only with the greatest difficulty make the proper additions and deductions that an accurate conclusion may be reached. How can the mind say to the eye, which reports accurately what it sees, "you are deceived. The light by which you see that tissue is a distorted light. Therefore the color you report is not the real color of the object as seen by daylight. It is not so red as you say it is. Therefore the inflammation is not so bad, hence the case is not so serious."

We seldom think of it, but there is literally no color. The sky is not blue, but the light makes it appear so. Light, falling upon particles of matter, is diffused, but space, where there is no matter which will hold light, is absolutely black. The silver moon is matter, and this matter reflects the light of the sun to us. Therefore the moon is visible. The green grass reflects to our eye the green rays which come to it from the sun. Speaking broadly, if it could not reflect the green rays and could not reflect any other, the grass would be black. Color, therefore, does not exist except as the waves of light are thrown back into the eye from the object upon which we look. Waves of a certain length have a certain color. The slowest rays are red, and the quickest visible rays are violet.

If an object absorbs and neutralises all the rays or waves except the very long ones, and these are thrown back to us, we say the object is red. All light and color and every wave has ceased but red. The application of this to diagnosis must be immediately apparent. If a tissue is examined under city gaslight, in which the spectrum shows that there is a large excess of red rays, then a tissue, which in daylight would be normal, would under gaslight, appear to be much redder than it really is. The eye accurately reports the excess of red color, and as inflammation increases the ability of a tissue to reflect red rays, the mind instantly says the tissue is inflamed, while as a matter of fact, it is not the tissue that is inflamed, but, to put it in a curious way, it is the light which is inflamed. It has an excess of red.

If a physician is examining tissues under a green light, the tissues have an abnormal and ghastly appearance. Green light, if thrown upon a red surface in sufficient purity, leaves the red without light; therefore, the red appears black. If green rays fall upon a surface which in daylight would be red, the surface cannot respond; therefore, it has no color, and, in the absence of color, it is black.

It is unnecessary to go further in this line of thought, as physicians are already fully aware of the difficulties of proper diagnosis with artificial light. There are, however, some phases of the subject which are not always given consideration. In the case of an examination of the blood to discover an anemic condition, it should be remembered that, if examination under artificial illuminants is made, with an illuminant giving an excess of red, the condition of the blood appears much better than it really is, and under an illuminant which is deficient in red, the apparent condition of the blood is much worse than it really is.

So important is accurate judgment to the physician and the surgeon that the adoption of acetylene, which is really daylight at night, in the operating rooms of hospitals is almost a necessity. The spectral similarity between acetylene and sunlight has only been recently brought to the attention of physicians, and its advantages were immediately recognised.

The ordinary portable lights like a house lamp, with a suitable reflector, have been adopted and brought into use by physicians who have seen the necessity of acetylene. As a matter of fact, small acetylene lamps of a portable character are in use in fifty-four out of the sixty public hospitals in New York city. The most eminent physicians connected with these hospitals have spoken of the value of acetylene in the highest terms. The investigation of the matter went no further than New York city, but the large proportion of hospitals in which acetylene is appreciated and in constant use was a surprise to the writer. These minor units do not meet the full requirements of the case. They are convenient because they are movable, and as an accessory to the system of hospital lighting have been proved valuable. There should, however, be in every hospital operating-room a complete system of acetylene illumination, proper reflectors should be provided, and whether the source of acetylene should be the modern house generator or cylinders, which are now used so extensively in railroad illumination, is a question which should be decided by local conditions. A perfect light for emergency operations at night is a desideratum of primary importance, and there should be no hesitation in working out a plan by which the hospital operating room should be given this nearest approach to daylight at night.

Acetylene is within the reach of every physician, and especially those in the country, as an individual household generator is now made by manufacturers in almost every city, which will produce acetylene for lighting an entire house, at a cost, candle power for candle power, which compares favorably with city gas at a dollar per thousand cubic feet. The apparatus and piping are not expensive and can be put into any house by a good plumber in two or three days, without disturbing furniture or walls. Over 150,000 individual installations are now located in country homes throughout the United States, so that its safety and utility are completely demonstrated. Many physicians have adopted acetylene as the common illuminant for their homes, and find it of inestimable value in their practice, and of great benefit to their patients.

Acetylene illumination is already recognised of immense value by dye houses, lithographers, artists and others who require an illuminant which will give them the ability to discriminate closely between different shades and colors, and men of the profession will not be slow to add to their equipment so simple an improvement.

157 MICHIGAN AVENUE.

